# Validation of a Methodology to Investigate Care Inequities for Transgender Patients

**DOI:** 10.5811/westjem.21279

**Published:** 2025-05-20

**Authors:** Kellyn Engstrom, Fernanda Bellolio, Molly Moore Jeffery, Sara C. Sutherland, Kayla P. Carpenter, Gia Jackson, Kristin Cole, Victor Chedid, Caroline J. Davidge-Pitts, Kharmene L. Sunga, Cesar Gonzalez, Caitlin S. Brown

**Affiliations:** * Mayo Clinic, Department of Pharmacy, Rochester, Minnesota; † Mayo Clinic, Department of Emergency Medicine, Rochester, Minnesota; ‡ Mayo Clinic, Department of Health Sciences Research, Division of Health Care Policy & Research, Rochester, Minnesota; § Mayo Clinic, Department of Quantitative Health Sciences, Rochester, Minnesota; || Mayo Clinic, Division of Gastroenterology and Hepatology, Rochester, Minnesota; # Mayo Clinic, Division of Endocrinology, Diabetes and Nutrition, Rochester, Minnesota; ¶ Mayo Clinic, Department of Psychology, Rochester, Minnesota

## Abstract

**Introduction:**

Pain is a common chief complaint in the emergency department (ED), and there are known disparities in the management of pain among racial/ethnic minorities, women, and older adults. Transgender and gender diverse (TGD) individuals comprise another under-represented patient population in emergency medicine and are also at risk of disparities in care. To measure and evaluate the magnitude of care inequities among TGD individuals, first we need to be able to accurately identify the right cohort and comparison groups. The primary objective of this study was to establish an accurate and generalizable process for identifying TGD patients through the electronic health record (EHR). Secondary objectives included creating and validating a method for matching and comparing of TGD patients to cisgender patients.

**Methods:**

This was a retrospective, observational cohort study that included patients presenting to Mayo Clinic EDs with a chief complaint of abdominal pain across four states (MN, WI, AZ, FL) between July 1, 2018–November 15, 2022. Patients ≥12 years of age were included. Patients’ sex assigned at birth and gender identity was extracted from the EHR via patient-provided registration fields. Two independent investigators independently reviewed each medical record of the identified TGD patient to validate the accuracy of pulled gender identity. Discrepancies were resolved by a third reviewer. Each transgender patient was matched to cisgender GBQ males (gay, bisexual, queer), cisgender LBQ (lesbian, bisexual, queer) females, cisgender heterosexual males, and cisgender heterosexual females using propensity score (PS) matching. We calculated the PS values using a multivariable logistic regression model where being transgender was the outcome, and covariates in the model included age, site, mental health history, and gastrointestinal history.

**Results:**

We initially identified 300 patients as TGD based on electronic data pull. An additional 1,000 patients were also included in the cohort for matching purposes. The agreement between electronic and manual review was 99.9%, and the kappa was 0.998 (95% confidence interval 0.994–1.000). We were able to match patients except for GBQ males due to low numbers. There is a significant difference in age between groups (*P* <0.001) with GBQ males being older than other groups.

**Conclusion:**

The methodology for identifying transgender and gender diverse patients in the EHR was accurate compared to manual review of gender identity. The TGD patients were able to be well matched, except to GBQ males. This provides a validated method to identify TGD patients in the EHR and further study disparities they may receive in care.

## INTRODUCTION

Lesbian, gay, bisexual, transgender and queer (LGBTQ) individuals are estimated to make up between 2.3–8.0% of the North American population.^1^ This group includes transgender and gender diverse (TGD) people, who account for 0.6% of the population, with higher prevalence among adolescents (1.2–4.1%).^2^

These TGD patients have greater chronic illness burden related to several domains of the social determinants of health, including societal marginalization and poverty; lack of access to healthcare, employment, and housing; and higher rates of mental illness, depression and substance use.^1^ The emergency department (ED) is a health resource for vulnerable underserved populations; however, a qualitative study demonstrated that TGD patients avoid ED care due to past negative experiences.^3^–^7^

To measure and evaluate the magnitude of care inequities among TGD, first we need to be able to accurately identify the right cohort and comparison groups. Currently, there is no established method for identifying and statistically comparing TGD patients to a relevant comparison cohort. Previous studies involving TGD patients had significant variability in their methods. Often matching was based on different combinations of gender identity, age, sex assigned at birth, and sociodemographic factors.^8^–^12^ Our objectives in this study were to establish an accurate and generalizable process for identifying TGD patients through the electronic health record (EHR) and create and validate methods for matching and comparing of TGD patients to cisgender, LGB, and heterosexual patients.

We focused on patients presenting with abdominal pain, a very frequent chief complaint in the ED and one that is difficult to diagnose and treat and may, therefore, be associated with care inequities. It is a condition of particular relevance to transgender people, as abdominal pain may be associated with gender-affirming surgery and gender-affirming hormone therapy.^3^ Pain is the most common chief complaint in the ED, comprising over 50% of all visits and representing 80% of all referrals from clinicians.^3^,^13^ Despite the well-documented disparities in pain management in the ED among other minority populations, there is a lack of published literature describing pain management among TGD patients.

Our objective was to establish an accurate and generalizable process for identifying TGD patients through the EHR. A secondary objective was creating and validating a method for matching and comparing of TGD patients to cisgender, LGB, and heterosexual patients.

## METHODS

### Study Design

A protocol was written prior to study start. Content experts from gastroenterology, psychology, and endocrinology reviewed and provided feedback on this proposal. This was a retrospective, observational cohort study including patients ≥12 years of age presenting to the ED with a chief complaint of abdominal pain between May 5, 2018–November 15, 2022. The study adheres to the Strengthening of the Reporting of Observational studies in Epidemiology (STROBE) guideline.^14^ The following elements of optimal retrospective chart review were followed: abstractor training; case selection criteria; variable definition; performance monitored; inter-rater reliability mentioned and tested; and medical record identified. We used sampling methods and received institutional review board approval for this research.

**Population Health Research Capsule**

What do we already know about this issue?

*Transgender and gender diverse (TGD) patients have greater chronic illness burden but avoid ED care due to past negative experiences.*

What was the research question?

*We sought to create an accurate and generalizable process for identifying TGD patients through the electronic health record (EHR).*

What was the major finding of the study?

*The agreement between electronic and manual review to identify TGD patients was 99.9%, and the kappa was 0.998 (95% CI 0.994–1.000).*

How does this improve population health?

*The EHR accurately identifies TGD patients, a first step in evaluating the magnitude of care inequities.*

### Setting and Participants

We included patients who presented to Mayo Clinic EDs in Minnesota, Florida, Arizona, and Mayo Clinic Health System: a total of 21 academic and community EDs. Patients who declined research authorization for medical record review were excluded. An electronic report was pulled from the EHR to identify patients who presented to the ED with abdominal pain as their chief complaint.

### Variables

We identified TGD patients in the EHR (Epic Systems Corporation, Verona, WI) using patient-provided registration data and information provided by surveys sent to patients before visits. In the patient registration data patients provide their gender (female/male/non-binary/choose not to disclose), and their sex at birth (female/male/uncertain/choose not to disclose). Based on this data, patients who reported their gender as non-binary and those whose reported sex at birth was male and gender was female (or vice-versa) were flagged as TGD patients. From the surveys sent to patients prior to visits we focused on the gender identity question, where patients are asked to select their gender identity from the following options: male, female, nonbinary or genderqueer, transgender male/female-to-male, transgender female/male-to-female, other, or choose not to disclose. Responding genderqueer, transgender male/female-to-male, or transgender female/male-to-female to this question also flagged TGD patients. Among the non-TGD/cisgender patients, we also used the sexual orientation question from the patient surveys to classify patients. Response options to this question were as follows: straight (not lesbian or gay); bisexual; lesbian or gay; pansexual; something else; or choose not to disclose. Cisgender patients were considered heterosexual if they responded “straight (not lesbian or gay)”, and cisgender lesbian, gay, bisexual, queer patients were identified if their sexual orientation was listed as bisexual, lesbian, or gay.

We electronically retrieved data on sex at birth, gender identity, age, mental health, and gastrointestinal (GI) disease history. History of mental health disorder was defined as having a diagnosis of anxiety, depression or schizophrenia, and GI disorder was defined as having a diagnosis of inflammatory bowel disease based on International Classification of Diseases 10^th^ Revision (ICD-10) codes (Supplemental Table 1).

### Measurements

**Figure 1:**
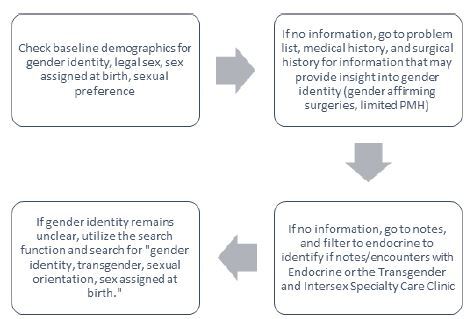
Gender and sexual identity verification method in electronic health record. *PMH*, past medical history.

Two independent investigators, blinded to each other’s responses, reviewed each medical record of the identified TGD patients to validate the accuracy of pulled gender identity (SS, KC, GJ). Data was verified by looking at patient demographic information and verifying that gender identity and sexual orientation matched the electronic extract. If there was no information available in the patient’s demographic information the patient’s problem list, medical history and surgical history were reviewed to identify gender-affirming surgeries or hormone therapy. If there was no information available, the chart was reviewed to identify any notes with endocrinology or the Transgender and Intersex Specialty Care Clinic. Lastly, if gender identity was still not identified, we used the search function within the EHR to search for “gender identity, transgender, sexual orientation, sex assigned at birth” (Figure 1). Investigators all underwent the same training for methods to manually identify gender identity. Discrepancies were reviewed by a third reviewer (KE) and resolved by consensus.

All other data variables were electronically extracted from the medical record, and 10% of the data was manually validated by an investigator (SS, KE, KC, GJ).

### Statistical Analysis

We summarized data using counts and percentages for categorical data, and medians and interquartile ranges for continuous data. Data were compared between the five groups using chi-square tests for categorical data and Kruskal-Wallis tests for continuous data. We calculated the percentage agreement and Kappa statistic to assess the agreement in identifying transgender patients using the electronic pull compared to manual chart review.

Each transgender patient was matched 1:1 matching where possible (one cohort did not have enough eligible visits to match all TGD patients) to cisgender male (gay, bisexual, queer) GBQ patients, cisgender female LBQ (lesbian, bisexual, queer) patients, cisgender heterosexual male patients, and cisgender heterosexual female patients using propensity score (PS) matching. We calculated a PS using a multivariable logistic regression model, where being transgender was the outcome and covariates in the model were age, site, mental health history, and GI history identified based on the diagnosis list of each patient (ICD-10). Patients were matched +/− 0.2 of the standard deviation of the logit of the PS.

## RESULTS

There were 43,191 patients with an ED encounter for abdominal pain during the study time frame, 25,527 of whom had provided information on sexual orientation and gender identity. A total of 300 identified as TGD. An additional 1,000 patients were also included in the cohort for matching purposes. Note that we matched a lower ratio of GBQ males due to small numbers of ED visits for abdominal pain in that group. A summary of the matching characteristics is shown in Table 1. There was a significant difference in age between groups (*P* <0.001) with GBQ males being older than other groups. Groups were otherwise able to be well matched based on hospital site, psychiatric history, and GI history. Upon manual review only one patient was found to have been incorrectly identified as TGD, this error occurred due to human error with demographic information entry in the EHR (Table 2). The agreement between electronic and manual review was 99.9%, and the kappa was 0.998 (95% CI 0.994–1.000). In the matching cohort an additional 302 patients had missing sexual orientation information. Despite missing sexual orientation information, the documented gender identity (LGBQ, heterosexual) information for these patients was sufficient to correctly categorize them for matching purposes. This information was added through verification of information from provider notes in the EHR, most being notes filed by primary care physicians (Figure 1).

## DISCUSSION

We sought to validate a method to identify TGD patients via an electronic data pull within our health system. Given the disparities in care received by TGD patients there is a growing need for study in this patient population to improve care and outcomes.^1^ Previously published retrospective literature varies on identification of TGD individuals. In their study Boyer and colleagues identified transgender individuals via ICD-10 codes.^10^ Another study by Abramovich et al identified transgender patients in the Canadian healthcare system via self-defined gender identify in the EHR.^15^

A study identifying thrombotic events in transgender patients receiving hormone therapy identified patients by including those whose sex was assigned as male at birth and receiving “feminizing drugs” and those whose sex was assigned female at birth receiving “masculinizing drugs.” Hormone treatment was determined through prescription data using national drug codes.^16^ However, none of these methods were manually validated to ensure the accuracy of identification of transgender individuals. Another method used to study TGD individuals is the Behavioral Risk Factor Surveillance System from the US Centers for Disease Control and Prevention.^17^,^18^ This survey collects data from United States residents regarding health-related risk behaviors, chronic health conditions, and use of preventative care. The results of this survey are limited to reported information and responses from individuals.

We found that an electronic data pull was accurate at identifying TGD patients in the EHR. Our results support the use of data extraction from the EHR for future TGD studies within our system to further identify disparities in the TGD population.

There have also been a variety of matching methods used in TGD studies. In a study by Pharr et al transgender patients were matched to cisgender patients 1:1 based on age, race, gender, income, education, and marital status; however, there were significant differences between the two groups.^8^ Another study by Boyer and colleagues matched transgender individuals to cisgender individuals by age and county.^10^ With our matching strategy we were able to match TGD patients with LBQ females, and heterosexual males and females, but we were only able to match 100 GBQ males in this cohort. There was also a significant difference in age with the GBQ males. Our matching strategy took into consideration the numerous sexual identities within a population and matched on baseline past medical history.

There are inherent limitations to retrospective studies. We found that the electronic data pull was accurate when compared to manual review of the gender identity in the EHR. However, we were unable to confirm with the patients themselves whether the documented gender identity and sexual orientation was accurate. Gender identity and sexual orientation are self-reported by patients and may lead to reporting bias with possible under-reporting. We were only able to match 100 GBQ males, compared to 300 in the LBQ females and heterosexual males and females. The GBQ male group was also older compared to our other four groups. It is likely secondary to lower rates of abdominal pain in this group. Lastly, accuracy of an electronic data pull may be variable based on different EHRs and documentation of gender identity.

## CONCLUSION

It is possible to accurately identify transgender and gender diverse patients using health records electronic data extraction tools. Continued research on the disparities TGD patients face is necessary so we can continue to improve the care of this patient population.

## Supplementary Information



## Figures and Tables

**Table 1:** Summary of matching patients.

	LGBQ Female (n=300)	LGBQ Male (n=100)	Heterosexual Female (n=300)	Heterosexual Male (n=300)	Transgender (n=300)	Total (N=1,300)	*P*-value
**Age**, Median (IQR)	24 (21, 31)	34 (27, 42)	24 (20, 31)	25 (20, 31)	24 (20, 31)	25 (20, 32)	<0.001
Site							0.11
ARZ	13(4.3%)	9(9.0%)	20(6.7%)	30(10.0%)	22(7.3%)	94(7.2%)	
FLA	15(5.0%)	11(11.0%)	19(6.3%)	21(7.0%)	18(6.0%)	84(6.5%)	
MCHS	195(65.0%)	52(52.0%)	179(59.7%)	157(52.3%)	173(57.7%)	756(58.2%)	
RST	77(25.7%)	28(28.0%)	82(27.3%)	92(30.7%)	87(29.0%)	366(28.2%)	
**History of anxiety**	203(67.7%)	64(64.0%)	200(66.7%)	184(61.3%)	197(65.7%)	848(65.2%)	0.53
**History of depression/bipolar**	214(71.3%)	64(64.0%)	212(70.7%)	188(62.7%)	207(69.0%)	885(68.1%)	0.12
**History of schizophrenia**	9(3.0%)	4(4.0%)	10(3.3%)	13(4.3%)	8(2.7%)	44(3.4%)	0.82
**History of somatic symptoms**	7(2.3%)	3(3.0%)	11(3.7%)	7(2.3%)	9(3.0%)	37(2.8%)	0.86
**History of IBD**	8(2.7%)	7(7.0%)	10(3.3%)	9(3.0%)	12(4.0%)	46(3.5%)	0.32

*LGB*, lesbian, gay, bi-sexual; *HS*, heterosexual; *ARZ*, Arizona; *FLA*, Florida; *MCHS*, Mayo Clinic Health System; *RST*, Rochester; *IBD*, inflammatory bowel disease.

**Table 2:** Validation of gender identify.

		Manual Review	
		Transgender	Other
Electronically Identified	Transgender	299	1
	Other	0	1000

Agreement: 99.9%

Kappa: 0.998 (95% CI 0.994–1.000)
