# Supplementary material for: Validation of a Methodology to Investigate Care Inequities for Transgender Patients
Source: West J Emerg Med. 2025 May 20;26(3):425–30. doi: 10.5811/westjem.21279 (PMC12208020; doi:10.5811/westjem.21279)
Supplement: Supplementary file 1 [file wjem-26-425-s001.docx]

**Supplemental Table 1.** International Classification of Diseases 9^th^ and 10^th^ revisions.

|  | ICD-10 codes | ICD-9 codes |
| --- | --- | --- |
| Anxiety | F40.8, F40.9, F41.1, F41.3, F41.8, F41.9, F06.4, F10.180, F10.280, F10.980, F12.180, F12.280, F12.980, F13.180, F13.280, F13.980, F14.180, F14.280, F14.980, F15.180, F15.280, F15.980, F16.180, F16.280, F16.980, F18.180, F18.280, F18.980, F19.180, F19.280, F19.980, F43.22, F43.23, F93.0 | 293.84, 293.89, 300.0X, 309.21 |
| Depression/bipolar | F06.31, F06.32, F25.0, F25.1, F30.X, F31.X, F32.X, F33.X, F34.X, F39, F41.8, F43.21, F43.23, F51.13, F53.0, F53.1, O96.6, Z13.31, Z13.32, Z86.59 | 296.X, 298.0, 306.8, 308.0, 309.0, 309.1, 309.4, 311, 313.1, 648.42, 648.44, V79.0 |
| Schizophrenia | F20.X, F84.5 | 295.X |
| Somatic symptoms | F45.X, F68.1X, F68.A | 300.X, 301.51, 307.89, |
| IBD | K50.00, K50.01X, K50.10, K50.11X, K50.80, K50.81X, K50.90, K50.91X, K51.00, K51.01X, K51.20, K51.21X, K51.30, K51.31X, K51.40, K51.41X, K51.50, K51.51X, K51.80, K51.81X, K51.90, K51.91X | 555.X, 556.X |

*IBD*, inflammatory bowel disease.
